# A scoping review of evaluation frameworks and their applicability to real-world physical activity and dietary change programme evaluation

**DOI:** 10.1186/s12889-020-09062-0

**Published:** 2020-06-26

**Authors:** Judith F. Fynn, Wendy Hardeman, Karen Milton, Andy P. Jones

**Affiliations:** 1 UKCRC Centre for Diet and Activity Research (CEDAR) and Norwich Medical School, University of East Anglia, Norwich, UK; 2 School of Health Sciences, University of East Anglia, Norwich, UK; 3 Norwich Medical School, University of East Anglia, Norwich, UK

**Keywords:** Programme evaluation, Framework, Physical activity, Dietary change, Scoping review

## Abstract

**Background:**

Physical activity and dietary change programmes play a central role in addressing public health priorities. Programme evaluation contributes to the evidence-base about these programmes; and helps justify and inform policy, programme and funding decisions. A range of evaluation frameworks have been published, but there is uncertainty about their usability and applicability to different programmes and evaluation objectives, and the extent to which they are appropriate for practitioner-led or researcher-led evaluation. This review appraises the frameworks that may be applicable to evaluation of physical activity and/or dietary change programmes, and develops a typology of the frameworks to help guide decision making by practitioners, commissioners and evaluators.

**Methods:**

A scoping review approach was used. This included a systematic search and consultation with evaluation experts to identify evaluation frameworks and to develop a set of evaluation components to appraise them. Data related to each framework’s general characteristics and components were extracted. This was used to construct a typology of the frameworks based on their intended programme type, evaluation objective and format. Each framework was then mapped against the evaluation components to generate an overview of the guidance included within each framework.

**Results:**

The review identified 71 frameworks. These were described variously in terms of purpose, content, or applicability to different programme contexts. The mapping of frameworks highlighted areas of overlap and strengths and limitations in the available guidance. Gaps within the frameworks which may warrant further development included guidance on participatory approaches, non-health and unanticipated outcomes, wider contextual and implementation factors, and sustainability.

**Conclusions:**

Our typology and mapping signpost to frameworks where guidance on specific components can be found, where there is overlap, and where there are gaps in the guidance. Practitioners and evaluators can use these to identify, agree upon and apply appropriate frameworks. Researchers can use them to identify evaluation components where there is already guidance available and where further development may be useful. This should help focus research efforts where it is most needed and promote the uptake and use of evaluation frameworks in practice to improve the quality of evaluation and reporting.

## Background

Programmes that aim to increase physical activity and improve dietary behaviours in individuals, groups and populations play a central role in addressing local, national and global public health priorities [1, 2]. Recent strategies have advocated approaches that are multi-sectorial, community-centred and evidence-based [1, 3–5]. Understanding if, when, and how these programmes are effective is important to justify policy, programme and funding decisions, and to inform and improve future decisions and practice. In order to achieve this, there is a need for appropriate and comprehensive programme evaluation [6, 7].

Practice-based evidence is generated from formal evaluation of programmes in real-world settings and is a fundamental part of evidence-based public health [8–10]. Those involved in the design, delivery and commissioning of physical activity and dietary change programmes are expected to evaluate programmes and contribute to the evidence base. However, real-world behaviour change programmes are complex and difficult to evaluate [11, 12]. The challenges of programme evaluation may relate to contextual factors that influence the complexity of the programme itself, e.g. its setting, target population, intervention function(s), or intended outcome(s) [12], or to factors that influence the evaluation priorities and objectives, e.g. differing stakeholder evaluation needs and organisational, political or resourcing factors [13]. Some of the practical challenges in conducting evaluation include the use of appropriate evaluation methods and tools, understanding what counts as evidence and how that is applied, and the roles of practitioners and researchers in evaluating real-world programmes [7, 9, 11, 14, 15].

Evaluation frameworks facilitate a systematic approach to evaluation and can help mitigate against some of the above challenges. Frameworks can enable multiple stakeholders to gain a shared understanding of the programme and evaluation process, and help to identify and agree upon appropriate objectives and methods. In this way, they can facilitate a more comprehensive evaluation, and may improve the fit between researcher-led and practitioner-led evaluation approaches [14]. A range of evaluation frameworks have been published. These include those developed specifically for use in programmes targeting specific health behaviours, conditions or populations (e.g. physical activity programmes [16–18]), those developed for health promotion and public health programmes more broadly (e.g. RE-AIM [19]), and generic frameworks intended to be applicable across a range of contexts, settings and sectors (e.g. Realist Evaluation [20]).

It is noteworthy that there is wide variation in the use of terminology used to describe frameworks, in the format of different frameworks, and in the context and ways in which they are intended to be used. Differentiating between frameworks, guidance, models or tools can be a challenge [21]. In this review the term ‘evaluation framework’ is used to include any structured guidance which facilitates a systematic evaluation of the implementation or outcomes of a programme. A ‘generic’ framework is used to refer to one that is intended for use across a range of contexts, settings and sectors, as opposed to one that has been developed for use in a specific context or field. Several frameworks have been developed for evaluation of programme implementation (process evaluation), whilst others focus on programme effectiveness (outcome evaluation) or are intended to facilitate an overall or comprehensive evaluation. In order to understand the content and focus of the frameworks and the contexts in which they may be applied, we have referred to the individual elements encompassed within evaluation as an “evaluation component”.

Many frameworks and developments in evaluation come from the research community, yet their intended audience and purpose is often unclear. For example, questions remain about the extent to which these frameworks are intended for use in practitioner-led or researcher-led evaluation, and their applicability to different evaluation objectives, programmes, and contexts.

Previous reviews of evaluation frameworks have been limited to frameworks which evaluate specific aspects of a programme, for example health inequalities [22], or methods used in health programme evaluations [23, 24]. Within the field of implementation science, reviews have focused on frameworks for translation of research to practice [25, 26]. The review by Denford et al. [27] made a valuable contribution by providing an overview of guidance available to support evaluation of public health programmes. However, it was limited to a subset of 48 documents created or sourced by national and international organisations and published since 2000. As a result some key evaluation frameworks published before 2000 or within the academic literature were not included, such as RE-AIM [19] and Realist Evaluation [20]. Denford et al. included various guidance documents intended for use in evaluating programmes targeting a broad range of health behaviours and health problems (e.g., smoking, asthma), as well as generic ones. Whilst they suggested that the wealth and breadth of available evaluation guidance may be a limiting factor in the ability of practitioners to access and apply appropriate guidance, the resulting review [27] and associated online catalogue [28] may still overwhelm practitioners seeking guidance on how to evaluate their specific programme.

To resolve some of this complexity we sought to develop a typology of frameworks, to help guide decision making by those involved in programme evaluation. The purpose was to appraise the frameworks that may be applicable for the evaluation of physical activity or dietary change programmes. By mapping the frameworks against a range of evaluation components (such as elements of process or outcome evaluation), we aimed to develop an overview of guidance included in each framework, enabling practitioners, commissioners and evaluators to identify and agree which frameworks may best meet their needs.

### Objectives

1. To identify published frameworks that can be used for evaluation of physical activity and/or dietary change programmes.

2. To identify each framework’s stated scope in order to assess their applicability to different evaluation objectives, programmes and contexts.

3. To identify and map which evaluation components are encompassed within each framework.

4. To use the findings to develop a typology of frameworks.

## Method

A scoping review approach was used, as this allowed the extent and nature of the literature on evaluation guidance to be identified and an overview of the available frameworks to be developed [29–31]. In line with the stages of a scoping review [29, 30], the process involved identification of the research question, a systematic search, consultation with experts, and mapping of the frameworks against different components of evaluation. We followed the PRISMA–ScR statement for the reporting of scoping reviews [32].

### Search strategy

To identify any frameworks that could be applied to physical activity and/or dietary change programmes, we used a broad search strategy to find those intended for use in public health, health promotion and generic programmes as well as those developed specifically for use in evaluating physical activity and dietary change programmes. Firstly, a search was conducted in Scopus. As a meta-database, including records from MEDLINE and EMBASE as well as other sources, Scopus is the world’s largest abstract and citation database of peer-reviewed literature. It contains sources across a range of fields including medicine, sciences, humanities and social sciences. The following search strategy was used: (TITLE ((framework OR model OR guid* OR tool)) AND TITLE-ABS-KEY ((“physical activity” OR exercise OR diet OR obes* OR overweight OR “public health” OR “health promotion”)) AND TITLE-ABS-KEY (communit*) AND TITLE-ABS-KEY (evaluat*)). No date restriction was applied. The search was undertaken in March 2018. All sources identified from the search were downloaded into the Endnote reference manager, and any duplicates were removed.

Secondly, between April and September 2018, we searched for grey literature on the websites of key organisations interested in evaluation of physical activity and/or dietary change programmes, using “evaluation framework” as a search term. This included the World Health Organization (WHO), Public Health England (PHE), Sport England, and the Centers for Disease Control and Prevention (CDC). Additional sources were identified from the authors’ existing files. We consulted evaluation experts and stakeholders including academics, those involved in public health policy development and evaluation, and evaluation consultants within the domains of physical activity or dietary change, to augment the search results. These experts were contacted and asked to provide feedback on the list of frameworks we had identified by the search strategy and to identify any omissions. Reference lists were examined for additional relevant sources.

Sources were screened by title and abstract, and then by full text (JF). Full text screening was independently validated (KM) and disagreements resolved through discussion. Consensus could not be reached for six sources, which were checked by a third reviewer (AJ) and agreed through further discussion.

### Inclusion and exclusion criteria

Inclusion and exclusion criteria were defined a priori and applied to all sources (JF). Table 1 provides details of the full inclusion and exclusion criteria. Sources were included from both the academic and grey literature that described a framework to support systematic evaluation of a physical activity and/or dietary change programme, including generic, public health or health promotion frameworks applicable to physical activity or dietary change programmes. Academic literature included journal articles and books. Grey literature was defined as all other printed and electronic documents published by organisations and agencies. Web-based sources were included if they provided systematic guidance on how to conduct an evaluation but excluded if they were an organisation’s general website without guidance. Only sources in English were included.

**Table 1:** Inclusion and Exclusion Criteria

Inclusion criteria	Exclusion criteria
Sources describing a framework or guidance to support evaluation of a programme e.g. process &/or outcome evaluation.	Sources describing a specific measurement tool.
Sources describing a framework or guidance to facilitate evaluation of physical activity, dietary change, public health or health promotion programmes.	Frameworks designed to support evaluation of programmes targeting other health behaviours (e.g. smoking, alcohol, substance abuse) or conditions not specifically linked to physical activity or dietary behaviours (e.g. HIV, mental health).
Sources describing a framework or guidance to support evaluation of a specific evaluation component that aligns with the underlying principles of real-world, community-based or health promotion programmes, e.g. community development, participation, wider health and non-health outcomes.	Sources describing frameworks or guidelines intended to support evaluation of technology-based programmes or cost-effectiveness, as these are related to distinct specialised areas of evaluation or health promotion approach.
Empirical and/or methodological studies reporting the development and/or validation of an evaluation framework, as well as conceptual or discussion papers describing a framework or guidance on evaluation.	Theoretical or conceptual models of conditions or interventions. Guidance on policy or action for management of disease, policy or clinical practices. Evaluation studies reporting the use of an evaluation framework.

### Data extraction and synthesis

To address the first and second objective, a data extraction template was used to collate information about each framework. The name of each framework was identified. Where no framework name was provided in the source, a short name was given based on the authors’ description in the title or abstract. To assess each framework’s scope and applicability to the evaluation of physical activity and/or dietary change programmes, data extraction fields included the stated evaluation objective, the types of programme it was intended for, and additional data related to general characteristics of each framework, e.g. its intended audience, format and development process.

To address the third objective we developed a set of data extraction fields to enable us to appraise whether each framework provided any guidance on a range of evaluation components, and what that guidance comprised. We have used the term ‘evaluation component’ to refer to individual elements encompassed within evaluation; for example elements of process or outcome evaluation. The list of evaluation components included in the data extraction template was identified a priori, and developed through a process of consensus building. We initially identified a list of evaluation components that were informed by recommendations for good practice in the evaluation literature, for example implementation, reach and unanticipated outcomes [12, 33–35]. This was further developed through consultation with evaluation experts, who were contacted and asked to comment on the appropriateness of the evaluation components we had identified and to identify any gaps or additional components based on their personal experience and knowledge of programme evaluation. Table 2 shows the full list of evaluation components grouped into those related to: (1) process evaluation, (2) outcome evaluation and (3) study design. Grouping programme context, theory of change and logic models within process evaluation components aligns with its inclusion in the UK Medical Research Council (MRC) Process Evaluation guidance [35], and recognises the crucial role of logic models in the early stages of developing an evaluation plan, in reporting causal assumptions about how a programme works, and informing process and outcome questions and methods. Where possible, pre-defined categorical responses were developed to facilitate the data extraction, coding and synthesis.

**Table 2:** Evaluation Components Agreed for Data Extraction and Mapping of Frameworks

Groups of evaluation components	Evaluation components for data extraction
(1) Process Evaluation	Describing programme context
	Using theory of change or logic models
	Reach
	Implementation
	Maintenance
	Any other process measures stated
(2) Outcome Evaluation	Behavioural outcomes
	Health outcomes
	Non-health outcomes
	Unanticipated outcomes
(3) Study Design	Stakeholder involvement
	Participatory evaluation
	Evaluation linked to stages of programme
	Evaluation at different time points
	Study design/method
	Data collection
	Data analysis
	Dissemination and reporting of findings

Where authors had described the scope of a framework variably, and where terms were not mutually exclusive, multiple terms were noted in the data extraction table. For example, terms such as community or practice based were used interchangeably to describe a study, intervention, setting or population. Where frameworks gave more detailed guidance on specific evaluation components, we also extracted a summary of what the guidance comprised. For each evaluation component we assessed whether the framework simply mentioned or provided more detailed guidance on how to evaluate or break down the relevant component.

Data extraction was completed by JF. To verify the data extraction, a random sample of 20 sources was checked independently by AJ and WH. Differences were resolved through discussion and used to establish agreed definitions that were then applied to further data extraction.

Framework format, programme type and evaluation objectives are typically used to describe frameworks. We therefore used these aspects to develop our typology for the frameworks. For the purposes of categorising the frameworks within the typology we used the dominant term presented in the description and content of the source as the basis for identifying each framework’s most defining characteristic. The extracted data was also used to map each framework against the evaluation components in order to provide an overview of the guidance encompassed within the frameworks. A narrative synthesis of the findings is presented.

## Results

### Study selection

**Fig. 1:**
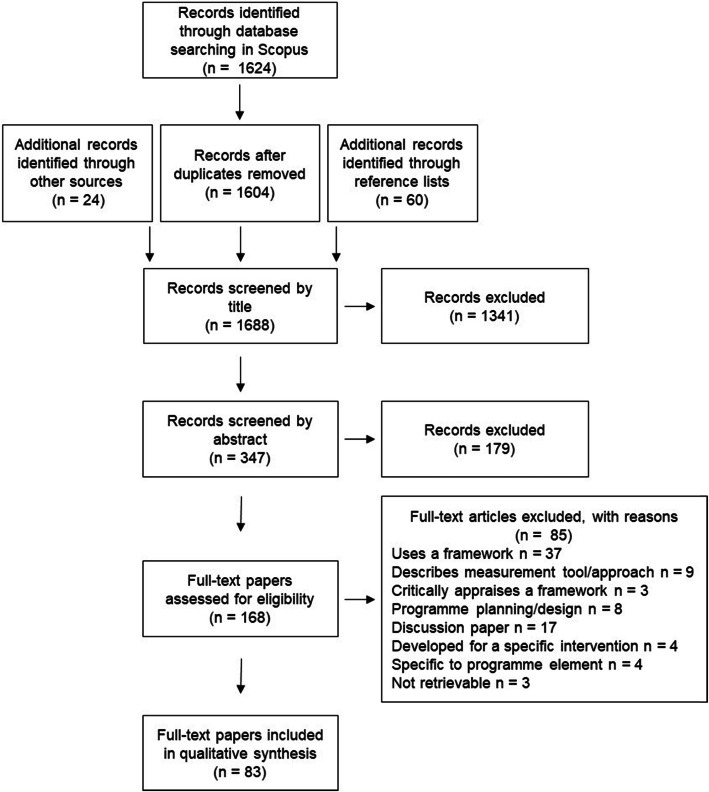
PRISMA diagram of screening process

The initial search in Scopus yielded 1604 sources once duplicates were removed. An additional 24 sources were identified from the grey literature search and consultation process, and a further 60 sources were identified from reference lists. Many articles were identified as ineligible from their title alone, mostly because they related to conceptual models, treatment models, or conditions not relevant to physical activity or diet. If there was any uncertainty regarding the potential eligibility of a paper, it was included in the next stage of the screening process. After screening of titles and abstracts 168 full-text sources were assessed for eligibility (PRISMA diagram, Fig. 1).

At full-text screening 83 sources were included and 85 were excluded. Of those excluded, 37 were reported evaluation studies that used one or more framework(s) and three were sources that critically appraised framework(s) [36–38]. The reference lists of these sources were searched to identify the index papers that described the frameworks mentioned.

Sources which described programme and evaluation practices in general terms, e.g. [39], and those which described a specific measurement tool, e.g. photovoice [40] and memorable messages [41] were excluded. Other sources were also excluded if they reported a framework linked to a specific intervention and in such a way that it was not generalisable (e.g. Framework for Washington State’s Healthy Communities Projects [42]). Planning frameworks that were solely for guidance on the design and development of an intervention were also excluded (e.g. [43–45], but a number were retained where they included guidance related to evaluation [46–50].

For frameworks which were described in more than one publication, for example in full and summary articles, we included both sources to facilitate data extraction and analysis, e.g. PRECEED-PROCEDE [46, 51], the CDC Framework [15, 52], UK MRC Guidance [12, 53–55], and Impact Pathway Analysis [56, 57]. Data were extracted from 83 sources, describing 71 evaluation frameworks.

### Identification of the evaluation frameworks available

A brief description of each framework is provided in Additional File 1 and an overview of their general characteristics is provided in Additional File 2. Table 3 lists the frameworks included in the review, grouped by decade of publication and source (academic/grey literature). All included frameworks were published during the last three decades (1990 onwards). Forty-two were described in academic publications and 23 in the grey literature. Six frameworks were reported in both the grey and academic literature [35, 52–57, 114–116].

**Table 3:** Included frameworks grouped by decade of publication and source

	1990–1999	2000–2009	2010–2018
Academic Literature^1^	*Evaluation of Health Education* [58] *Evaluation of Healthy Community Initiatives* [59] *Health Workers Guide* [60] *Realistic Evaluation* [20] *Utilization-Focused Evaluation* [61] Framework for Outcome Assessment [62] Intervention Mapping [47, 63] MMIPP [64] PRECEDE-PROCEED [46, 51] Stages of Evaluation Model [33, 65] **Principles for Evaluating Community HP** [66] **RE-AIM** [19]	*California Healthy Cities Framework* [67] *Setting Standards* [34] Community Initiative Evaluation Model [68, 69] Evaluation in Health Promotion [70] Formative Model of Service Evaluation [71] Planning, Implementation & Evaluation Model [72] Process Evaluation for Public Health [73] Six Step Guide to Process Evaluation [74] **Concepts in process evaluation** [75] **Evaluating Legacy of community health initiatives** [76] **Getting to Outcomes** [77] **HEBS Framework** [14] **Levels of Coalition Evaluation** [78] **Participative Framework Health Inequalities** [79] **Participation, Partnerships & Equity** [80] **Settings for Health Promotion** [49] **Well Connected** [81]	*Cross-site Evaluation Tool* [82] *Empowerment Framework in Nutrition* [83] *Evaluating Complex Community-Based HP* [84] Generic Evaluation Toolkit [85] Systematic Evaluation Multiple Components [86] **Contextual Factors Framework** [50] **Coordinated Action Checklist** [87] **Multilevel Framework** [88] **OPEN tool** [89] **Process Evaluation in Group Settings** [90] **Process Evaluation Cluster Randomised Trials** [91] **Supportive Social Environments for Health** [92] **Three Dimensional Health Cube** [93]
Grey Literature^1^	*WHO Recommendations* [94]	*Kellogg Foundation Evaluation Handbook* [95] *Logic Model Development Guide* [48] *NICE Guidance: Behaviour Change* [96] Evaluating Community Projects [97] Framework for Community Health [98] Evaluating Sport & Physical Activity [99] Health Planners Toolkit [100] LEAP [101] Physical Activity Evaluation Handbook [102] Sport England Evaluation Framework [103] **SEF for Weight Management** [18]	Better Evaluation [104] Centre TRT’s Framework [105] Community Toolbox [106] Evaluation Works: a toolkit [107] Public Health England (PHE) Guide [108] Magenta Book [109] Ontario Evaluation Workbook [110] Victoria Govt DoH Framework [111] **GPAT** [112] **SEF for Dietary Interventions** [17] **SEF for Physical Activity** [16]
Both	CDC Framework [15, 52]	*MRC Complex Intervention Guidance* [53, 54] Impact Pathway Analysis (PIPA) [56, 57, 113]	*MRC Process Evaluation Guidance* [12, 35, 55] *MRC Natural Experiments* [114, 115] **GENIE** [116, 117]

*Italics = flexible guidance*, Normal text = frameworks formatted as steps, Bold = frameworks formatted as a set of indicators

^1^Academic literature included journal articles and books. Grey literature was defined as all other printed and electronic documents published by organisations and agencies

Table 3 also indicates the format of each framework. This ranged from highly structured to more flexible guidance. Thirty of the frameworks were presented as a set of steps; typically, these steps align with the stages of programme development and implementation. Twenty-four frameworks were presented as a set of indicators or questions, ranging from those that included a small number of key indicators [19, 79, 81, 93] to those that encompassed a longer checklist of evaluation criteria or questions [16–18, 112, 117]. The remaining 17 provided flexible evaluation guidance.

Sources generally described the framework development as being based on (i) some combination of literature review, consultation and testing, (ii) experiences of conducting evaluation(s), or (iii) prior frameworks or theory. Many of the more recently published frameworks referred to earlier ones as informing their development, such as realist evaluation [20], utilization-focused evaluation [61], PRECEDE-PROCEED [46] and intervention mapping [63]. Several frameworks formatted as a set of steps mentioned the CDC framework [52] and other step-based frameworks [73, 75] as informing their development. Several frameworks formatted as a checklist referred to RE-AIM [19] as informing the indicators.

Seventeen frameworks provided guidance or links to sources for additional support or training in using the framework. Those that gave more detailed guidance of training and support, including links to additional resources, were predominantly published within the grey literature and had an online presence [95, 101, 103, 104, 107].

### Scope of the evaluation frameworks and development of a typology

There was considerable heterogeneity in the terminology used to describe the scope of the frameworks. Authors described them variously in terms of purpose, content, or applicability to different programme and/or evaluation contexts. Additional File 2 shows the range of the descriptors used by authors. For example, thirty-one sources mentioned the frameworks were intended for use in real world or practice-based settings, and 22 were intended for use in community-based programmes, with these terms often used interchangeably. Others were described as applicable to specific intervention functions (e.g. health education [117] or policy [77, 94, 105]), or specific intervention or study types (e.g. complex interventions [44, 54, 84], natural experiments [114] or cluster randomised trials [91]). These terms were not mutually exclusive so were not used to categorise the frameworks and develop the typology but are indicated within Additional file 2.

### Programme type

**Fig. 2:**
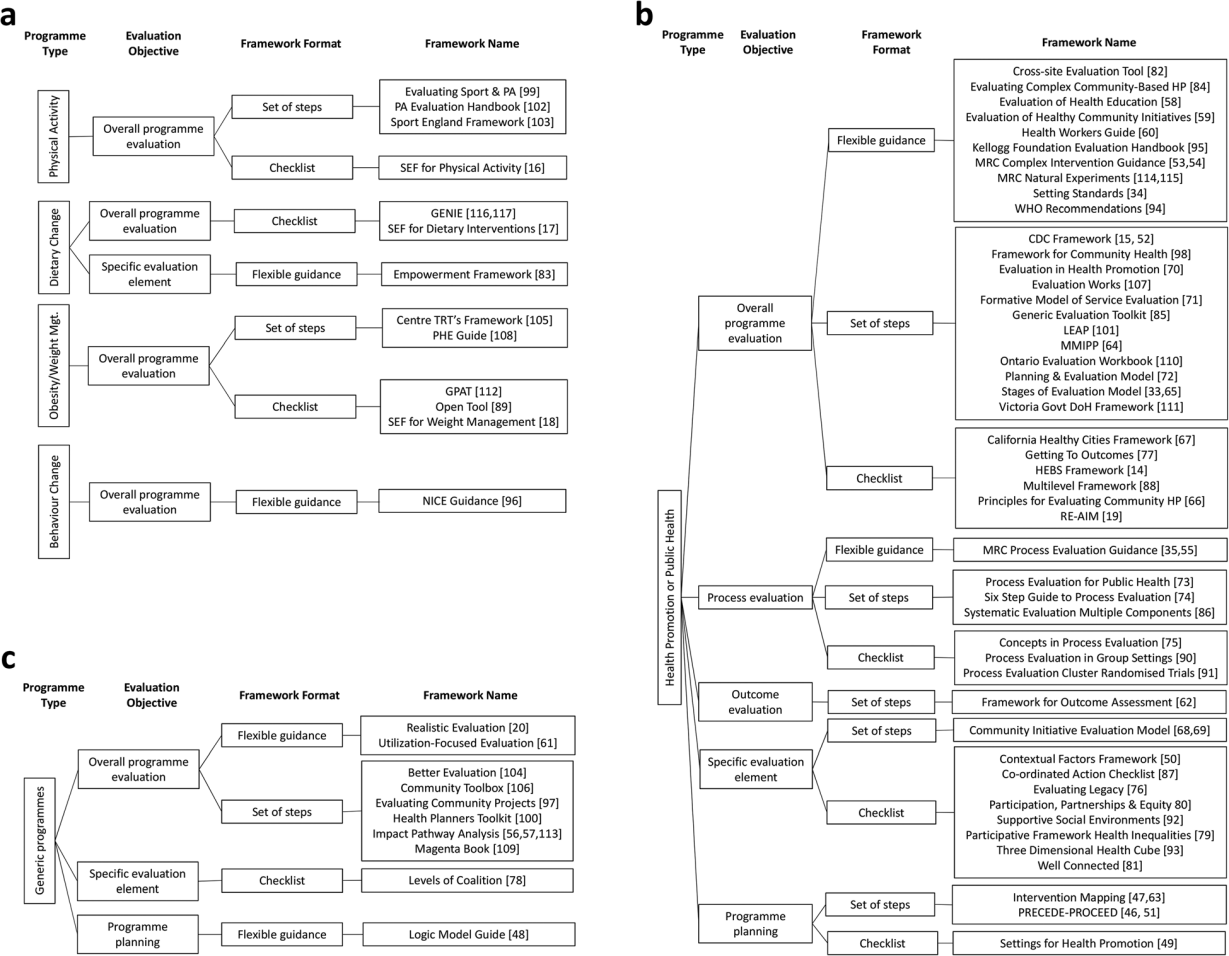
**a** Typology of evaluation frameworks intended for use in physical activity, dietary change or behaviour change programmes. **b** Typology of frameworks intended for use in health promotion or public health programmes. **c** Typology of frameworks intended for use in generic programmes

Despite this variability in descriptors used by authors, we used the intended programme type as the primary categorisation to develop the typology, followed by the evaluation objective and the framework format. These characteristics enabled us to group the frameworks by applying the dominant description provided by the authors as an indication of a framework’s most defining characteristics. Figures 2a-c show the typology which signposts to each framework within the categories.

Twelve frameworks were stated as intended for use in physical activity and/or dietary change programme evaluation, and one as for use in behaviour change interventions [96] (Fig. 2a). Forty-eight were described as for use in public health or health promotion programmes. Some of these clearly stated how their components related to health promotion principles. However, several used the terms health promotion and public health interchangeably, and these were therefore grouped together (Fig. 2b). A further ten frameworks were described as applicable to a range of programme types and we have grouped these as intended for generic programme evaluation (Fig. 2c).

### Evaluation objective

Frameworks were also described variously in terms of their evaluation focus or objective, and we used this to further develop the typology shown in Figs. 2a-c. Fifty-two were stated as providing guidance on overall programme evaluation, nine as specific to process evaluation and one as specific to outcome evaluation. Several of the frameworks provided guidance on evaluating specific programme elements such as empowerment [83], partnerships and participation [68, 78, 80, 81, 87, 92], contextual factors [50], or legacy [76]. Four frameworks were described as ‘planning frameworks’ but incorporated guidance on evaluation [46–49]; these are grouped separately within the typology (Figs. 2a-c). Other frameworks that included guidance to facilitate both evaluation and planning, but were not specifically described as ‘planning frameworks’, e.g. [50] are not grouped separately.

### Mapping frameworks against evaluation components

Frameworks were mapped against seven process and four outcome evaluation components (i.e. describing programme context, using theory of change, logic models, reach, implementation, maintenance, any other process measures, behaviour, health, non-health and unanticipated outcomes), as well as against the eight components of study design and reporting (see Table 2). Tables 4a-c and 5a-c provide an overview of the mapping. Describing programme context, theory of change, and logic models are crucial to informing process and outcome evaluation, we therefore included these alongside process evaluation components in Table 4a-c. The mapping enabled us to develop an overview of the guidance included in each of the frameworks and appraise their applicability to different evaluation objectives and to physical activity and/or dietary change programmes.

**Table 4:**
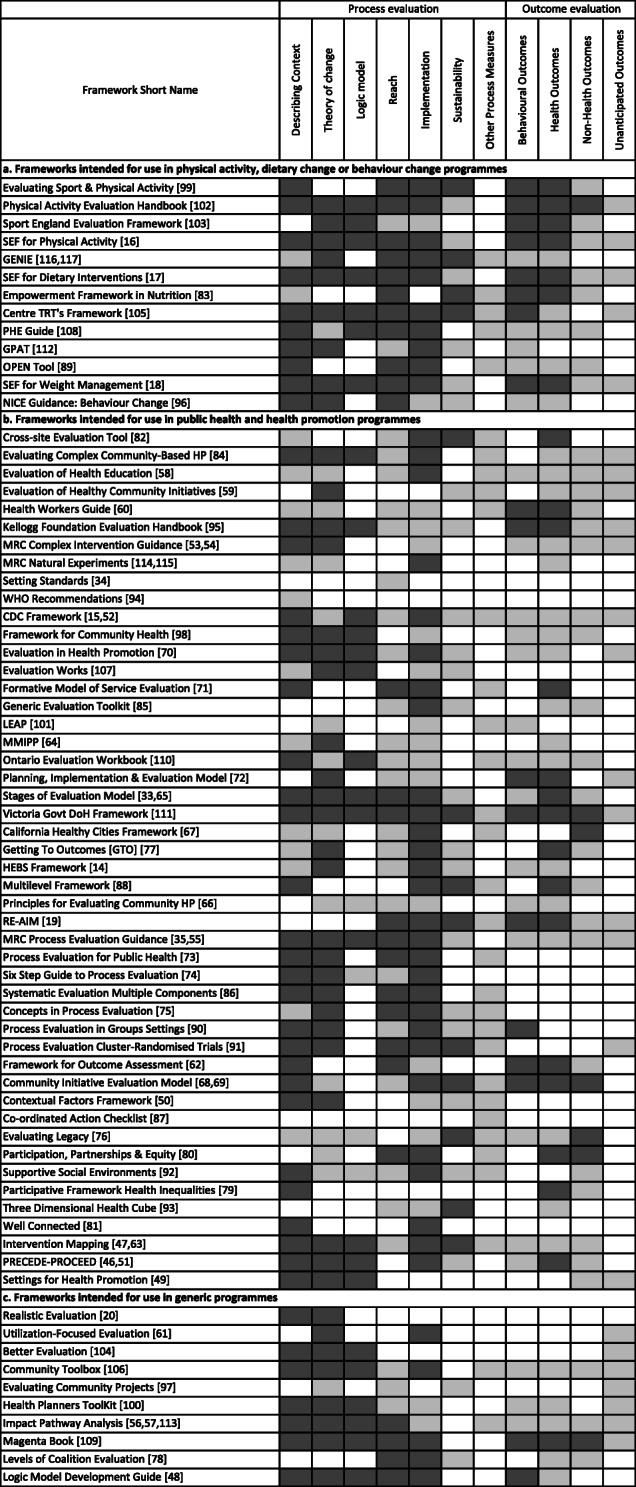
a Frameworks intended for use in physical activity, dietary change or behaviour change programmes mapped against process and outcome evaluation components. Light grey shading indicates the component is mentioned, dark grey shading indicates more detailed guidance on how to break down or evaluate the component. b. Frameworks intended for use in evaluating public health and health promotion programmes mapped against process and outcome evaluation components. Light grey shading indicates the component is mentioned, dark grey shading indicates more detailed guidance on how to break down or evaluate the component. c Frameworks intended for use in evaluating generic programmes mapped against process and outcome evaluation components. Light grey shading indicates the component is mentioned, dark grey shading indicates more detailed guidance on how to break down or evaluate the component.

**Table 5:**
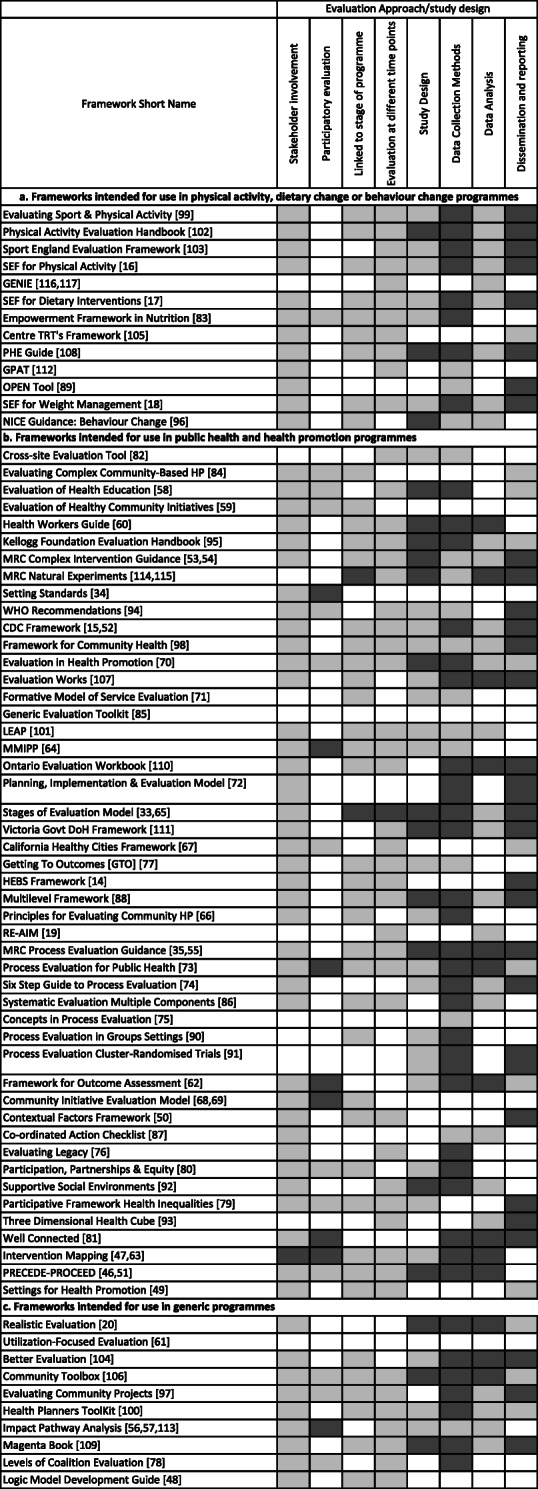
a Frameworks intended for use in evaluating physical activity, dietary change or behaviour change programmes mapped against study design, evaluation approach and reporting components. Light grey shading indicates the component is mentioned, dark grey shading indicates more detailed guidance on how to break down or evaluate the component. b Frameworks intended for use in evaluating public health and health promotion programmes mapped against study design, evaluation approach and reporting components. Light grey shading indicates the component is mentioned, dark grey shading indicates more detailed guidance on how to break down or evaluate the component. c Frameworks intended for use in evaluating generic programmes mapped against study design, evaluation approach and reporting components. Light grey shading indicates the component is mentioned, dark grey shading indicates more detailed guidance on how to break down or evaluate the component.

Many frameworks mentioned components without any further details (shaded in light grey in the tables), whilst others provided detailed descriptions of how the components may be broken down or evaluated (shaded in dark grey in the tables). For ease of navigation, the frameworks in Tables 4a-c and 5a-c are grouped and listed in the same order as in the typology (Figs. 2a-c). Most frameworks included guidance on a range of both process and outcome evaluation components. Eleven frameworks did not provide any guidance on outcome evaluation and were specific to process evaluation e.g. [73–75, 81]. Frameworks intended to facilitate evaluation of specific programme elements focused on a narrower range of components that aligned with their stated purpose [50, 76, 78, 80, 93].

### Process evaluation components

Guidance on the key components of process evaluation were included in most frameworks, e.g. describing contextual factors of programmes, identifying and describing causal mechanisms or theories of change, reach and implementation. The frameworks providing the most comprehensive and detailed guidance on these components include the MRC guidance on process evaluation of complex interventions [12], Center of Excellence for Training and Research Translation (Center TRT) Framework [105], Victoria Government Department of Health (DoH) Evaluation Framework [111], the Physical Activity Evaluation Handbook [102] and the Standard Evaluation Frameworks (SEFs) [16–18]. Other process evaluation components were included within fewer frameworks. For example, guidance on evaluation of sustainability was limited, with only thirteen frameworks providing more details of how to evaluate it, e.g. [76, 93]. A small number of frameworks mentioned other process components such as adaptation, exposure, capacities, training, partnerships, satisfaction, and community changes; however, details of how to evaluate these components were limited. Over half the frameworks identified logic models as a useful tool in programme planning and evaluation. Several of these provide more detailed information, examples and/or templates to support the development of logic models [12, 48, 95, 108].

### Outcome evaluation components

Guidance on outcome evaluation components was more variable than for process evaluation components. Frameworks designed for use in physical activity and/or dietary change related programmes provided more detailed information on evaluation of behavioural and health outcomes than the more generic evaluation frameworks. Evaluation of non-health outcomes was typically only mentioned briefly in the frameworks, with only seven providing any level of detail [67, 68, 76, 80, 102, 109, 111]. Only about one third of the frameworks mentioned evaluation of unanticipated outcomes, and none provided further information on how to evaluate them.

### Study design components

Tables 5a-c shows the frameworks mapped against components related to study design, including evaluation at different time points, stakeholder involvement, participatory approaches, data collection and analysis, and reporting of findings. Most frameworks identified the importance of stakeholder involvement and/or participatory evaluation approaches. Few provided information on how to incorporate this, with a few exceptions that did provide detailed guidance on participatory evaluation methods [56, 57, 68, 69, 79].

Most frameworks mentioned the importance of conducting evaluation that is appropriate to a programme’s stage of development, and many were presented as a set of steps aligned to stages of programme development and implementation. Most also mentioned evaluation at different time points (i.e. baseline and follow-up), mainly in relation to outcome measures only. Several frameworks used the terms formative and summative evaluation but gave limited information on how they were defining them, or how to do these types of evaluation. Exceptions to this were frameworks that gave a more detailed explanation of the role of formative and pilot studies in developing an intervention [33, 53].

Guidance on data collection and data analysis was highly variable. Several frameworks provided explanations of appropriate use of experimental designs and quantitative and qualitative methods [20, 46, 54, 75]. Others provided more detailed guidance on specific data collection methods and measures [16–18, 33, 72, 86, 100, 110]. Only thirteen frameworks provided information to guide data analysis. There was more consistency in the inclusion of guidance on data collection and analysis within the frameworks described as specific to physical activity and/or dietary change programmes than in the other categories of frameworks.

Finally, guidance on dissemination and reporting also varied. Many frameworks mentioned the importance of this aspect within the cycle of evidence-based practice, but few provided information about where and how to report findings to different target audiences.

## Discussion

Our scoping review identified 71 evaluation frameworks, considerably more than previous reviews of evaluation frameworks within the field of public health [25–27]. The broad search strategy we applied enabled us to identify frameworks developed within a range of domains that we could add to those included in these earlier reviews. The focused set of inclusion and exclusion criteria we then applied meant that we only included frameworks specific to or generalisable to physical activity and/or dietary change programmes. In addition to the 12 frameworks specifically intended for physical activity and/or dietary change programme evaluation, we identified a further 59 intended for public health, health promotion, behaviour change or generic programmes that were applicable to physical activity and/or dietary change programmes.

Our review has highlighted the plethora of frameworks available; previous reviews [27] reported this as a potential challenge to practitioners and evaluators navigating and making use of the available guidance. Our review also highlighted the variability in terms used by authors to describe the purpose and scope of the frameworks. Although we identified a growing number of frameworks developed by and for practitioners, e.g. [102, 103, 106, 107, 111], in many frameworks the intended audience was unclear. Terms used to describe programme types were poorly defined and were often used interchangeably. Some phrases such as ‘natural experiment’ and ‘real-world’ were used to refer to the evaluation approach and the intervention itself, whilst others (e.g. behaviour change and sustainability) were used to refer to both intervention processes and outcomes. Several frameworks which stated they were intended to support both programme planning and evaluation provided insufficient details about how these facilitated evaluation. The lack of clarity in the extent to which frameworks are intended to be used by researcher-led or practitioner-led evaluation, and in their applicability to different programmes and evaluation objectives, has implications for those using the available guidance. There needs to be a greater consensus of how terms are defined within public health evaluation. An agreed common language would enable those involved in programme evaluation to understand more clearly the applicability of the different frameworks and would help this research area to move forward.

Our typology and mapping resolves some of that complexity in purpose and scope of frameworks by signposting to relevant frameworks and by developing an overview of what guidance is encompassed within each. Our appraisal of frameworks has highlighted areas of overlap, strengths and limitations in the guidance available to support programme evaluation. For example, the inclusion of key process evaluation components (e.g. describing programme contexts and causal mechanisms, reach, and use of logic models) in most frameworks reflects the growing understanding of the importance of these aspects of evaluation to facilitate a more detailed understanding of whether and how a programme works [7, 33–35, 118]. These components represent strengths within the existing guidance, and areas where there is already an abundance of guidance.

The mapping process and appraisal also identified components where more guidance would be beneficial. We found limited guidance on participatory approaches, non-health and unanticipated outcomes, and wider programme components (e.g. resources, training, delivery, adaptation, partnerships, organisational structures), and sustainability. These components represent aspects of evaluation that require further development of guidance. Stakeholder involvement or participatory evaluation was mentioned in all but nine of the frameworks, reflecting the growing recognition of the importance of stakeholder engagement in evaluation decisions and processes [34, 84]. However, detailed guidance on how to incorporate participatory evaluation methods was only provided by seven frameworks [34, 56, 64, 68, 73, 80, 81], and represents another area where further development of guidance would be beneficial. Compared to other categories within the typology, frameworks specific to physical activity programmes more consistently provided guidance on evaluation of health and behavioural outcomes, including the use of appropriate data collection and analysis methods. By their nature these components are specific and therefore may be difficult to define within more generic frameworks. Frameworks developed to facilitate evaluation of specific programme elements, such as sustainability [76, 93], and those intended to facilitate evaluation of partnerships [78, 80, 92] or community [68, 69, 80] also addressed some of the gaps within the more generic frameworks.

Our mapping and typology signpost to frameworks where guidance on specific components can be found. Although availability does not necessarily equate to accessibility or usability of information, the mapping of frameworks can be used to help understand some of the strengths and limitations within the guidance provided. Further investigation of whether and how frameworks have been used may provide insight into how fit for purpose they are, and the benefits and challenges of applying them within physical activity or dietary change programme evaluation. Furthermore, the typology and mapping can be used by practitioners, commissioners and evaluators of physical activity and/or dietary change programmes to identify frameworks relevant to their evaluation needs. They can also be used by researchers and those interested in developing evaluation guidance to identify evaluation components where it would be most useful to focus their efforts, rather than developing more guidance for components where there is already an abundance of guidance. Our categorisation could also be used by researchers publishing frameworks to more clearly report how these are intended to be used, and for those reporting evaluation studies to more clearly state how they have been used.

### Strengths and limitations

Our broad search strategy enabled a comprehensive review which identified 71 frameworks within the academic and grey literature. By drawing on frameworks developed within different domains, we have added to previous reviews [25, 27] to map a wide range of evaluation frameworks applicable to physical activity and/or dietary change programmes.

Our scoping review methods, which included consultation with experts, helped to maximise the chances of identifying relevant frameworks, and of applying relevant components which were based on consensus to appraise the frameworks. It was not our intention to apply a formal consensus building method, however we recognise that the use of a more formalised process would be an alternative approach. By consulting both practice and research-based experts we are confident that the results will be of interest and value to both practitioners and researchers concerned with evaluation of physical activity and/or dietary change programmes.

There are limitations of the review. The review only included sources published in the English language. The heterogeneity and ambiguity in use of terminology was a methodological challenge during screening, data extraction and synthesis. Frameworks intended to support specialist evaluation aspects such as health economic evaluation and evaluation of programmes using digital technologies (e.g., mobile health) are critical to practice and policy decisions, however we excluded these frameworks due to their specificity and also due to the large number available. A separate review of the available guidance to support these specialist evaluation aspects would be beneficial.

## Conclusion

We have added to previous reviews of evaluation frameworks, and identified 71 frameworks applicable to physical activity and/or dietary change programme evaluation. There is an abundance of frameworks available to support programme evaluation. Our typology and mapping signpost to frameworks where guidance on specific components can be found, where there is overlap in their scope and content, and where there are gaps in the guidance. Practitioners and evaluators can use the typology and mapping to identify, agree upon and apply appropriate frameworks. Researchers who develop evaluation guidance can use them to identify evaluation components for which there are gaps in available guidance. This should help focus research efforts where it is most needed and promote uptake and use of appropriate evaluation frameworks in practice to improve the quality of evaluation and reporting.

## Supplementary information

**Additional File 1.** Brief descriptions of the frameworks.xlsx. A short summary of each framework based on the stated scope and aim provided by the original authors of each framework.

**Additional File 2.** General characteristics of the frameworks.xlsx. Summary of data extracted to identify the scope and general characteristics of each framework.

## Data Availability

All data generated or analysed during this study are included in this published article [and its supplementary information files]. All data used in this manuscript is in the public domain.

## Abbreviations

CDC: Center for Disease Control and Prevention
Center TRT: Center of Excellence for Training and Research Translation
DoH: Department of Health
GPAT: Good Practice Appraisal Tool
HEBS: Health Education Board Scotland
HP: Health Promotion
LEAP: Learning, Evaluation and Planning
MRC: Medical Research Council
MMIPP: Model for Management of Intervention Programme Planning
NICE: National Institute for Health and Care Excellence
OPEN: Obesity Prevention through the European Network
PIP: Programme Impact Pathway
PIPA: Participatory Impact Pathway Analysis
PA: Physical Activity
PH: Public Health
PHE: Public Health England
RE-AIM: Reach, Efficacy, Adoption, Implementation, Maintenance
SEF: Standard Evaluation Framework
WHO: World Health Organization
